# Finger Millet (*Eleusine coracana*) Plant–Endophyte Dynamics: Plant Growth, Nutrient Uptake, and Zinc Biofortification

**DOI:** 10.3390/microorganisms11040973

**Published:** 2023-04-08

**Authors:** Renu Chaudhary, Vijay Kumar, Sanjay Gupta, Bindu Naik, Ram Prasad, Sadhna Mishra, Per Erik Joakim Saris, Vivek Kumar

**Affiliations:** 1 Himalayan School of Biosciences, Swami Rama Himalayan University, Jolly Grant, Dehradun 248016, India; renuchoudhary2512@gmail.com (R.C.); vijaykumar@srhu.edu.in (V.K.); sanjaygupta@srhu.edu.in (S.G.); 2 Department of Life Sciences, Graphic Era (Deemed to be) University, Bell Road, Clement Town, Dehradun 248002, India; binnaik@gmail.com; 3 Department of Botany, School of Life Sciences, Mahatma Gandhi Central University, Motihari 845401, India; rpjnu2001@gmail.com; 4 Faculty of Agricultural Sciences, GLA University, Mathura 281406, India; sadhnamishra2649@gmail.com; 5 Department of Microbiology, Faculty of Agriculture and Forestry, University of Helsinki, 00100 Helsinki, Finland

**Keywords:** endophyte, finger millet, zinc, biofortification, nutrient uptake, plant growth promotion

## Abstract

Endophytic fungi and bacteria were isolated from finger millet and their effects on finger millet growth parameters and zinc and NPK contents in grains were studied. Out of 70 fungal and 112 bacterial endophytes, the two best fungal and bacterial isolates were selected on the basis of zinc solubilization and plant-growth-promoting attributes. The fungal isolates identified were *Aspergillus terreus* and *Lecanicillium* sp., and the bacterial isolates were *Pseudomonas bijieensis* and *Priestia megaterium*. The endophytic zinc, NPK mobilization, and plant-growth-promoting efficacy were determined in a pot experiment with zinc carbonate as the zinc source. Endophytic-primed plants showed enhanced shoot and root lengths compared to the unprimed control. Endophytes increased the zinc content in grains by between 12.12% and 18.80% compared to control plants. Endophytes also augmented the NPK concentrations in seeds compared to control plants and exhibited stability in a diverse range of pHs, temperatures, and NaCl concentrations, and exhibited growth on various carbohydrate and nitrogen sources. This is the first study reporting the interaction of *Aspergillus terreus*, *Lecanicillium* sp., *Pseudomonas bijieensis*, and *Priestia megaterium* with finger millet for grain Zn biofortification and NPK concentration enhancement. This study indicated that zinc-dissolving endophytes possess the potential for enhancing the zinc and NPK content in grains in addition to the plant-growth-promoting attributes.

## 1. Introduction

The issues related to the injudicious application of chemical fertilizer in farming have led to growing attention to the use of beneficial endophytic microorganisms to enhance plant health and crop production while safeguarding food quality and environmental viability. Finger millet (*Eleusine coracana*) is considered to be a nutrition-rich crop, is used as food and feed in Asian and African nations, and is regarded as a staple food for the underprivileged [1,2]. With a global production of roughly 4.5 million metric tons, finger millet is the sixth most significant cereal crop. With 1.2 million metric tons produced, India comes in first place behind Africa’s 2.5 million metric tons [3]. Finger millet is a significant crop in the semiarid and arid regions of Asia and Africa and it is adaptable to various agro-climatic conditions [4]. Additionally, finger millet is more dense in nutrients than rice and wheat, since millet grains are rich in zinc, iron, phosphorus, calcium, potassium, magnesium, carbohydrates, vitamins, and vital amino acids. They are also particularly high in calcium and polyphenols [2,5,6]. It has nutritious components that are simple to digest, making it a staple food for diabetics, childbearing women, the ill, nursing mothers, and children [7].

Healthy soil plays an important role in the sustainability of planet earth, since healthy soil is rich in macro- and micro-nutrients and contains a plethora of microbes. Crops growing on such soils should provide an adequate amount of nutrients; consumers relying on such healthy crops should not have nutrient insufficiencies [8]. Ref. [9] emphasized the importance of soil, soil science, and soil scientists in achieving the Sustainable Development Goals of the United Nations very effectively. A crucial tactic for recovering the benefits, products, and resources that ecosystems provide to humanity is the rehabilitation and restoration of land. In order to sustain or restore the functions of the local ecosystem, soil solutions strive to improve soil health. Sustainability can be achieved by increasing soil moisture and decreasing droughts and soil erosion [10]. Furthermore, when considering the local climate and soil conditions, soil health represents the ability of the soil to deliver ecosystem services in a specific area. A healthy and sustainable landscape also includes soil and the water that enters the soil and contains sediments and nutrients [11]. However, it is important to resolve zinc deficiencies in an environmentally friendly and sustainable way to ensure enduring soil productivity and health. The Zn availability in Indian soils may be as low as 47% [12]; furthermore, the zinc availability in arable land with a low pH is deemed to be poor [13].

Zinc, one of the important micronutrients, is essential in small amounts for the normal development and growth of plants and humans. Due to its crucial functions in biological processes, Zn is an important constituent in the human diet. Zn is essential for healthy cellular function, including differentiation, cell division, growth, and transport within the cell; endocrine and immunological systems; transcription; synthesis of proteins and nucleic acids; and DNA replication. Additionally, Zn plays a role in the sense of taste and helps healthy growth and development during the prenatal period, infancy, childhood, and adolescence [14]. Zinc deficiency may lead to the risk of developing a range of diseases and unfavorable functional impairments, including blindness, cognitive decline, lowered IQ, stunting, preterm birth, and a greater risk of infection during pregnancy [15].

In crop plants, Zn plays a vital antioxidant role and specifically participates in the metabolism of carbohydrates and auxin. Crops with zinc deficiencies have fading leaves and diminutive development. Consumption of zinc-deficient millet increases the probability of zinc deficiency in humans as well [16]. The unavailability of soluble zinc in the soil or lack of access to the plant is a major reason for zinc deficiency [17]. Although plants are capable of absorbing zinc as a divalent cation, the amount of soluble zinc in soil solution is lower, and the remaining zinc is found as insoluble minerals and compounds [18]. Various methods have been adopted to solve zinc deficiency, such as the use of chemical fertilizers; conventional, transgenic, or breeding approaches; and so on. An appealing alternative could be the usage of efficient endophytes capable of dissolving zinc in soil into available forms to meet plant Zn needs.

In recent years, the use of endophytes as bioinoculants to enhance agricultural productivity has been suggested as an efficient substitute to boost crop productivity [19]. Endophytes are vital to the growth and adaptation of plants to varied ecological circumstances at every stage of their development. Plant-growth-promoting endophytes stimulate plant growth by producing phytohormones and act as biocontrol agents to protect plants from various diseases or by solubilizing nutrients and aiding in nutrient uptake. Acidification is one of the methods used by microorganisms to dissolve zinc. Organic acids are secreted into the soil, and bind Zn cations and lower the pH of the surrounding soil [17]. Additionally, the anions have the ability to chelate zinc and increase its solubility. Other potential pathways for the solubilization of zinc include siderophore and proton generation, oxidoreductive systems in cell membranes, and chelated ligands [20].

Many studies have revealed that endophytes can dissolve zinc, which can be taken up by crop plants [21,22]. The amazing capacity of Zn-dissolving bacteria to enhance the Zn content in a crop’s edible parts has been investigated [23,24]. Zn-dissolving endophytes improve Zn localization in the edible parts of chickpeas [25,26], rice [27], wheat [28], and maize by *Microbacterium* sp. [29], and could be an effective biofortifying agent. 

Considering the aforementioned information, this study was planned to isolate, identify, and exemplify the endophytic fungi and bacteria from finger millet for their potential to mobilize and transport zinc in plants, increase NPK uptake, and promote host growth.

## 2. Materials and Methods

Procurement of finger millet seeds:

In this study, seeds of three cultivars of the finger millet -PRM-1, VL-348, and VL-352 were procured from VCSG Uttarakhand University of Horticulture and Forestry, Bharsar, Uttarakhand, India.

### 2.1. Isolation of Endophytic Microbes

Seeds were sown in the research fields of VCSG Uttarakhand University of Horticulture and Forestry, Bharsar, Uttarakhand, India (30.30574° N, 78.9924° E), and healthy finger millet plants were selected for endophyte isolation. To remove dirt particles, plant samples were first rinsed in tap water, then 6–7 times in sterile distilled water. A further surface sterilization process was carried out by separating the stem, leaves, and root portion of the fledgling finger millet plant. Surface sterilization of plant samples was performed using 70% ethanol for one minute, then for two minutes, using 2% sodium hypochlorite on leaf and stem surfaces, and 2% sodium hypochlorite for three minutes on root surfaces. After six sterile distilled water rinses, they were then dried in laminar flow. A 100 µL aliquot of the final wash was spread on a nutritional agar and potato dextrose agar medium to confirm sterility using the pour plate method and incubated for 5 days at 28 °C for a bacterial sterility check and 7 days at 25 °C for a fungal sterility check. If no growth was observed on the plates after incubation, the surface sterilization process was deemed successful, and the endophyte isolation process was resumed; otherwise, the entire process of surface sterilization was redone. In order to isolate endophytes, plant tissue samples were mashed separately (stem, leaves, and root) with a sterilized mortar and pestle to create a uniform paste, which was then allowed to settle for 20 min. The supernatant was further serially diluted in 12.5 mM phosphate buffer (pH 7.1), and 100 µL of each (10^−1^ and 10^−2^) was spread on nutrient agar and minimal agar g/L (dextrose, 1.0; dipotassium phosphate, 7.0; monopotassium phosphate, 2.0; sodium citrate, 0.5; magnesium sulfate, 0.1; ammonium sulfate, 1.0; agar, 15.0; pH, 7.0), and incubated for seven days at 28 °C. Potato dextrose agar plates were incubated for 10 days at 25 °C. For further studies, endophytic fungi and bacteria were isolated, purified on the basis of distinct colony characteristics, and stored on a slant. This procedure was performed for all three cultivars.

### 2.2. Selection of Effective Zinc Solubilizing Endophytes

The qualitative screening for the selection of prospective Zn solubilizing endophytic fungi and bacteria was performed by following the scheme [30]. Tris minimal medium containing (g/L) 6.06 g Tris–HCl; 4.68 g NaCl; 1.49 g KCl; 1.07 g NH_4_Cl; 0.43 g Na_2_SO_4_; 0.2 g MgCl_2_·2H_2_O; 30 mg CaCl_2_·2H_2_O; and 15 g agar, pH 7.0 was doped separately with 0.1% of different zinc sources, i.e., zinc oxide, zinc carbonate, and zinc phosphate. Prior to use, all glassware was soaked in 0.1 M HNO_3_ for an hour, then washed three times in distilled deionized water. Tris minimal agar medium plates were spot inoculated by freshly grown endophytes (fungi and bacteria) isolated on potato dextrose agar and nutrient agar medium, then incubated for seven days at 28 and 25 °C, respectively. The clear halo zone formation around the colonies indicated zinc solubilization, the diameter (mm) of the halo zone and colony was determined and the solubilization index (mm) was determined as described in [31].

Zinc solubilization index (mm) = Colony diameter (mm)  +  Halo zone diameter/colony diameter (mm)

Plant growth-promoting attributes of selected zinc solubilizing endophytes

### 2.3. Indole Acetic Acid Synthesis

Selected zinc solubilizing fungal and bacterial endophytes were examined for their capability to synthesize indole acetic acid (IAA). The endophytic cultures grown for 24 h were inoculated in nutrient broth supplemented with L-tryptophan (1.0 mg mL^−1^) and incubated for 48 h at 28 ± 2 °C in a shaking incubator at 150 rpm. An amount of 5 mL of each culture was taken out of the broth and centrifuged for 30 min at 4 °C at 6000 rpm. Then, 2 mL of Salkowski reagent (1 mL of 0.5 M, FeCl3 solution, 50 mL of 35% of perchloric acid) [32] was mixed with 1 mL of the supernatant and incubated for 30 min in dark; IAA production was indicated by pink color development. The optical density of the colored solution was measured at 530 nm using a spectrophotometer (SHIMADZU, model no. UV1800ENG240VSOFT) as μg mL^−1^, and a standard IAA graph was prepared to calculate the amount of IAA produced by endophytes, with uninoculated nutrient broth serving as a control.

### 2.4. HCN Production

#### 2.4.1. Qualitative Assay

The potential of endophytes to produce hydrogen cyanide was evaluated by the method presented in [33]. Endophytic fungi and bacteria were cultured on nutrient agar amended with 4.4 g/L glycine. A sterile Whatman filter paper was saturated in a 0.5% solution of picric acid and was placed inside the lid of a cultured Petri dish. Lids were covered using Parafilm and Petri dishes were incubated for 48 h at 25 °C. The synthesis of HCN was visually assessed based on the filter paper’s color change from deep yellow to dark brown and recorded as negative, weakly positive, moderately positive, and strongly positive, which were denoted as −, +, ++, and +++, respectively.

#### 2.4.2. Quantitative Assay

An evaluation of HCN production by endophytes was carried out by inoculation of each bacterial and fungal suspension in King’s B broth medium supplemented with glycine (4.4 g/L). Each tested isolate was duplicated three times. Flasks that were not inoculated served as the control. Sterile filter paper strips were dipped in an alkaline picrate suspension (0.5% picric acid and 2% Na_2_CO_3_) and tied to the flask’s neck (one strip for each flask). The flasks were plugged and covered with parafilm and incubated at 28 °C for four days at 140 rpm. In direct proportion to the amount of HCN produced, the color of sodium picrate strips changed to a reddish color. By adding the fresh filter paper to a test tube with 10 mL of deionized water, the color was eluted, and the color absorbance was measured using a spectrophotometer (SHIMADZU, model no. UV1800ENG240VSOFT) at 625 nm. Prior to measuring, the zero-absorbance reading at 625 nm was adjusted using distilled water [34].

### 2.5. Ammonia Excretion

For measuring ammonia production, cultures grown for 24 h were inoculated in peptone broth and incubated at 25 °C for 48 h. To each tube, 0.5 mL of Nessler’s reagent was added, and the appearance of a brown-to-yellow tint indicated the formation of ammonia [35]. 

### 2.6. Siderophore Production

#### 2.6.1. Qualitative Assay

A modified CAS agar medium in [36] was used to evaluate the ability of endophytic strains to produce siderophore-type chemicals that chelate iron. All glassware was washed in deionized water after being rinsed in 3 mol/L HCl to eliminate iron prior to the experiment. In brief, 121 mg of CAS was suspended in 100 mL of deionized water and 20 mL of a solution of 1 mM FeCl_3_.6H_2_O (ferric chloride) made in 10 mM HCl. This mixture was gradually added with stirring to a 20 mL solution of hexadecyl trimethyl ammonium bromide (HDTMA, 729 mg of HDTMA was suspended in 400 mL of deionized water.) Before further usage, the CAS-HDTMA suspension was sterilized. An amount of 100 mL of the CAS reagent was added to 900 mL of sterile LB agar medium for CAS agar plates preparation, and freshly grown bacterial and fungal culture spots inoculated on CAS agar in medium Petri dishes were incubated at 28 °C for 5–7 days. Petri dishes showing an orange-colored zone development around the colonies indicated siderophore production [37].

#### 2.6.2. Quantitative Assay

Bacterial cultures grown for 48 h in LB broth and fungal cultures in MSM (minimal salt medium broth) were used for quantitative siderophore production. One milliliter of broth (one for each bacterial and fungal culture) was placed in a 1.5 mL sterilized centrifuge tube, which was then sterilized before being inoculated with 10 µL of a freshly produced bacterial and fungal culture (10^8^ cfu/mL). For each strain, three replicates (tubes) were used. In addition, a control tube (uninoculated broth) was produced. Cultures of endophytic isolates were centrifuged at 10,000 rpm for 10 min after being incubated for 48 h at 28 °C; the supernatant was utilized to measure the siderophore levels. Each culture’s supernatant (0.5 mL) was mixed with 0.5 mL of CAS mixture, and after 20 min, the optical density at 630 nm was measured using a spectrophotometer (SHIMADZU, UV1800ENG240VSOFT). The amount of siderophore that strains produced was assessed in percent (%) siderophore units (psu), which was determined by means of the formula given in [38]: percent siderophore unit = Ar − As/Ar × 100, where the reference absorbance was Ar (CAS solution and uninoculated broth) at 630 nm and the sample absorbance was As (CAS solution and sample supernatant) [37].

### 2.7. Phosphate Solubilization

NBRI-BPB agar medium [39] was used to test the endophytic isolates’ ability to dissolve phosphate. The medium containing bromophenol blue is denoted as NBRI-BPB, and contains (g/L) 10-glucose, 5-Ca_3_(PO_4_), 5-MgCl_2_.6H_2_O, 0.25-MgSO_4_.7H_2_O, 0.2-KCl, 0.1-(NH_4_)_2_SO_4_, 0.025-BPB, and 15-agar pH (7.0) prior to medium sterilization. Freshly grown bacterial and fungal isolates were spotted on the NBRI-BPB agar plates and kept at 28 °C for 7 days and observed for halo zone formation around the colony, which is an indicator of phosphate solvatiom by the endophytic isolates. The solubilization index (mm) was calculated after measuring the halo zone and colony diameter (mm) by adapting a method in [31]. The colony and halo zone diameter were used to calculate the solubilization index.

### 2.8. Organic Acid Production

All selected zinc solubilizers were screened for organic acid production based on pH change using an agar plate assay. For this, each bacterial and fungal isolate was spotted on Czapek–Dox agar medium plates containing (g/L) 30.0 g sucrose, 0.01 g FeSO_4_.7H_2_O, 0.5 g KCl, 1.0 g KH_2_PO_4_, 0.5 g MgSO_4_, 2.0 g NaNO_3_, 15.0 g agar, 1000 µL/L triton X-100, and bromocresol green (pH indicator). The pH was adjusted to 6.0 before autoclaving the medium, and after inoculation, culture plates were kept for 4–7 days at 25 °C and observed for yellow zone formation around the colonies, an indicator of organic acid production [40].

### 2.9. Extra Cellular Enzyme Assay

Endophytic fungal and bacterial overnight cultures were spotted onto 1% Tween-20 Luria Bertani agar plates to measure lipase synthesis [41]; skimmed milk agar Petri dishes [42] were utilized for the determination of protease. Dishes were kept for 3–4 days at 28 °C to test for lipase and protease after being spot-inoculated with fresh fungal and bacterial cultures. Clear areas around colonies showed the corresponding activity. The amylotic activity was assessed using glucose yeast extract peptone agar medium appended with 0.2% starch solution and kept for 3–5 days at 25 °C [43]. An amount of 5 mL of 1% iodine solution was placed onto each Petri dish after the incubation period to measure the enzyme activity. The isolates were spotted onto urea agar slants and kept for 24 h at 37 °C to observe changes to reddish pink color, indicating positive urease activity [44]. Phytase screening media containing sodium phytate were used for phytase screening [45]. Gelatin agar (semisolid with 7.5 g/L agar) tubes were stabbed to test for the formation of gelatinase. The cultures were incubated for 48 h before being put in a refrigerator at 4 °C until the bottom resolidified. If gelatin was hydrolyzed, the medium will continue to be liquid even after cooling. The medium will resolidify in the fridge if gelatin was not hydrolyzed before use [46]. 

For detecting tannase enzyme synthesis, tannic acid was added to the tannic acid agar medium as a carbon source. The tannic acid was sterilized separately through a nitrocellulose membrane filter and the dishes were kept at room temperature (28 °C) for five days, after which, the plates were flooded with 0.01 M FeCl_3_, which reacted with metabolites to produce a brown color, an indicator of tannase production [47].

### 2.10. Stress Tolerance of Zinc Solubilizers

The selected fungal and bacterial endophytic isolates were tested for their tolerance to various salts, temperatures, and pH stresses by exposing the isolates to various pH ranges (5.0, 6.0, 7.0, 8.0, 9.0, and 10.0), different NaCl concentrations (1%, 2%, 3%, 4%, 5%, 6%, 7%, 8%, 9%, and 10%), and different temperatures (5 °C, 15 °C, 25 °C, 30 °C, 35 °C, 40 °C, and 50 °C). Culture plates were spot inoculated with freshly grown fungal and bacterial endophytic culture and incubated at 28 °C for 48 h. After incubation, the growth of isolates was recorded as +, ++, +++, and ++++.

### 2.11. Identification of Selected Zinc Solubilizers

#### 2.11.1. Morphological and Biochemical Characterization

The morphology of the colony, as well as biochemical and molecular studies, were used to characterize endophytic isolates.

##### Substrate Utilization Tests

The carbon and nitrogen utilization ability of isolates was assessed by using a basal medium containing (NH_4_)_2_SO_4_, 2.64 g/L; KH_2_PO_4_, 2.38 g/L; K_2_HPO_4_, 5.65 g/L; MgSO_4_, 1 g/L; CuSO_4_, 6.4 mg; FeSO_4_, 1.1 mg; MnCl_2_, 7.9 mg; ZnSO_4_, 1.5 mg; and agar, 15 g/L. The carbon utilization media contained 1% of various carbon sources, i.e., glucose, fructose, lactose, inositol, maltose, malt extract, sorbitol, mannitol, galactose, arabinose, rhamnose, ribose, trehalose, sucrose, mannose, and dextrin. Nitrogen utilization was tested by adding glucose (10 g/L) as a carbon source and 2.64 g/L of different types of nitrogen sources one by one into the above-mentioned medium. The pH was adjusted to 6.8–7.0 before autoclaving and cultured plates were incubated at 24–48 h at 28 °C and growth of endophytes was recorded as +, ++, +++, and ++++.

##### 16S rRNA Sequencing

Genomic DNA was extracted from the endophytic fungi as per a procedure defined previously in [48]. The amplification of ITS sequences was performed by using the primers ITS1: TCCGTRSGNGAACYTGHGG and ITS4: TCCTCCGCTTATTKATDTGC as described in a previous study [40]. Genomic DNA was prepared according to the method mentioned in [49]. The 16S gene amplification was performed by using universal primers 16F27-5′-AGAGTTTGATCMTGGCTCAG-3′ and R-1492R-5′-ACGG(CT)TACCTTGTTACGACTT-3′. The amplified products were resolved on 1% agarose gel and visualized under a gel documentation system (GelDoc UVPGelDoc-Ite imager). The PCR products of ITS (fungal) and 16S (bacterial) were sequenced by using the same primers by an ABI3500 genetic analyzer (Applied Biosystems, Waltham, MA, USA). The sequences were deposited to NCBI Genbank and the accession numbers were obtained. For phylogenetic and molecular analyses, MEGA X package software (Version 11) was utilized [50]. Using the CLUSTAL W software (Version 2) described in [51], the ITS sequences of the fungal-type strains retrieved from GenBank and bacterial sequences retrieved from the EZbiocloud database were matched with the corresponding type of fungal and bacterial nucleotide sequences, respectively. The Kimura 2-parameter model was used to examine evolutionary history using the maximum likelihood method [52]. The evolutionary history of the examined species is represented by the consensus bootstrap tree created from 1000 replicates. 

Additionally, the sequences of the chosen isolates were examined using the BLAST (Blastn) search engine and added to the GenBank database (NCBI). Based on maximal identity and habitat, the most acceptable relative sequences from the NCBI database were chosen and loaded into MEGA 5.0. By combining the neighbor-joining method with a bootstrap analysis using 1000 iterations, we created a rooted phylogenetic tree that intuitively revealed the genetic distances between various bacterial strains. 

### 2.12. Plant Experiment

On the basis of their zinc solubilization indices and plant-growth-promoting attributes, two fungal isolates, EC1F-15 and EC1F-23, and two bacterial isolates, EC3B-22 and EC3B-23, were selected for further study. Zinc carbonate (ZnCO3) was utilized as the zinc source in the pot experiment. The earthen pot experiment was conducted in four-liter pots filled with sterile soil. For inoculation, 3 mL of the bacterial culture suspension containing 10^8^–10^9^ CFU/mL and 2 mL of fungal spore (1 × 10^4^) suspension were used for inoculating the surface sterilized finger millet seeds and placed at the same depth (1 cm from topsoil) in all earthen pots. The control treatment consisted of sterile distilled-water-treated seeds (without bacteria/fungi). All the pots were arranged in a completely randomized blocked design with three treatments and control [53]. The following treatments were carried out to study the zinc uptake in finger millet plants, control (uninoculated), zinc carbonate (ZC), ZC + EC3B-22, ZC + EC3B-23, ZC + EC1F-15, and ZC + EC1F-23. Similar sets were made for all three cultivars (PRM1, VL348, and VL352), which were conducted in triplicates. The plants were cultivated for 90 days and were periodically irrigated with tap water. The growth parameters, such as root shoot length, dried biomass, and zinc uptake in seeds, were assessed.

### 2.13. Nutrient Analysis

The dried finger millet seeds were ground into an amorphous powder using a clean mortar and pestle, and 100 mg was added to a 150 mL Erlenmeyer flask containing 10 mL HNO_3_ (9:4 ratios with perchloric acid (HClO_4_)). The flasks were heated to 300 °C on a hot plate, where the seed materials were digested until the solution became completely colorless. The volume of the extract was transferred to a volumetric flask and, using deionized water, the final volume was adjusted to 100 mL. The zinc content of these samples was measured using an atomic absorption spectrophotometer (Electronics Corporation of India Ltd. AAS 4141).

For determining N content, seed extracts were prepared by digesting 0.5 g dried seeds first with concentrated H_2_SO_4_ and then HClO_4_, as described in [54]. The P concentration in the extract was ascertained colorimetrically using the molybdovanadate reagent method mentioned in [55], and the K concentration in the acid digested seeds was revealed directly by using the flame photometer method as described in [55].

### 2.14. Statistical Analysis

All experimental sets used a completely randomized block design (CRBD) for the microbial application on finger millet plants in order to detect the impact of PGPE. Duncan’s multiple range tests (DMRTs) were used to compare the difference between the mean values of different parameters at *p* < 0.05. Plant growth parameters were compared using ANOVA and Duncan’s means comparison. Data were analyzed using SPSS (version 22) for statistical analyses and Microsoft Excel (Window 11) was used to prepare graphs.

## 3. Results

### 3.1. Isolation of Endophytes

A total of 70 fungal and 112 bacterial endophytes were isolated from all three cultivars of the finger millet plant parts (root/shoot/leaves and seeds). These isolates were purified and named according to the crop variety name and were picked on the basis of distinct morphology types for further characterization and identification.

### 3.2. Endophytes Selection on the Basis of Zinc Solubilization

The zinc solubilization capability of the endophytic isolates was determined by the halo zone diameter. During the preliminary screening, out of seventy endophytic fungal isolates, ten isolates were selected on the basis of zone formation on Tris minimal medium agar plates containing zinc salts. Two prominent fungal isolates, i.e., EC1F-15 and EC1F-23, were selected for further study on the basis of the zinc solubilization index (SI). Both the selected isolates showed a halo zone on all three salts of zinc; EC1F-15 showed a maximum solubilization index of 3.0 mm for zinc carbonate followed by zinc oxide and zinc phosphate with solubilization indeces of 2.9 mm and 2.6 mm, respectively. EC1F-23 dissolved zinc oxide with a maximum solubilization index of 3.6 mm followed by zinc carbonate at 3.3 mm and zinc phosphate at 3.1 mm. Similarly, out of 112 endophytic bacterial isolates, two potent isolates, i.e., EC3B-22 and EC3B-23, were selected for further experiments. EC3B-22 and EC3B-23 both showed halo zones in zinc carbonate-, zinc oxide-, and zinc phosphate-amended medium, EC3B-23 created a maximum zone in ZnO, ZnCO_3_, and ZnPO_4_ with the SI equal to 5.6 mm, 4.8 mm, and 2.3 mm, respectively, while EC3B-22 dissolved ZnCO_3_, ZnO, and ZnPO_4_ with an SI of 4.3 mm, 3.4 mm, and 2.1 mm, respectively. For zinc carbonate, the maximum halo zone was formed by EC3B-23 (4.8 mm), followed by EC3B-22 (4.3 mm). For ZnO, EC3B-23 exhibited the highest solubilization index of 5.6 mm. Zinc phosphate was utilized by both isolates. Table 1 shows the solubilization index of the selected bacterial isolates.

#### Phosphate Solubilization

The phosphate solubilization potential of zinc-solubilizing endophytes was determined by using the agar plate method. The potential of endophytes to turn insoluble phosphate into the soluble form was evaluated by observing clear zones around the colony in NBRI-BPB agar media. Both fungal isolates EC1F-15 and EC3F-23 dissolved phosphate and showed solubilization indeces of 4.3 and 3.6 mm, respectively. Similarly, endophytic bacteria EC3B-22 and EC3B-23 dissolved phosphate with solubilization indeces of 2.4 and 2.5 mm, respectively (Table 1).

### 3.3. Plant Growth-Promoting Attributes of Selected Zinc Solubilizers

#### 3.3.1. IAA Production

For a qualitative assessment of IAA, all Zn solubilizing isolates showed color changes varying from light pink to reddish upon the adding Salkowski’s mixture, signifying hormone production. Fungal isolates EC1F-23 and EC1F-15 produced IAA (82.4 µg mL^−1^ and 35.4 µg mL^−1^, respectively). Among the bacterial isolates, both EC3B-22 and EC3B-23 synthesized IAA (117.2 µg mL^−1^ and 105.5 µg mL^−1^, respectively) in the presence of tryptophan (Table 2).

#### 3.3.2. HCN Production

In a qualitative analysis of HCN produced by fungal isolates, only EC1F-15 was able to change the color of the filter paper; further quantitative analyses of this endophyte showed a maximum absorbance of 0.031, while EC1F-23 displayed an absorbance of 0.009 (Table 1 and Table 2). Both the bacterial endophytes were able to produce HCN and showed a positive result in varying amounts; in qualitative analyses, EC3B-22 showed strong (+++) HCN production, while moderate production (++) was exhibited by EC3B-23. The isolates were further quantified spectrophotometrically, a quantitative analysis of the HCN produced by bacterial isolates showed that EC3B-22 produced a significant amount of HCN and showed the highest absorbance value of 0.043, followed by EC3B-23 with a value of 0.029 (Table 2).

#### 3.3.3. Ammonia Production

All four endophytes were screened for ammonia production, and only bacterial isolate EC3B-23 demonstrated ammonia production after adding Nessler’s reagent; the rest of the endophytes did not produce ammonia (Table 2).

#### 3.3.4. Determination of Siderophore Production

A positive siderophore synthesis is indicated by an orange color zone formation around the colony grown under limiting iron conditions by the CAS agar method. For the fungal endophytes, both showed siderophore production and exhibited an orange zone around the colony. In a quantitative estimation of siderophore production, fungal endophytes EC1F-15 and EC1F-23 produced 63.7 and 60.6 psu, respectively. The bacterial isolates EC3B-22 and EC3B-23 also showed orange zone formation around the colony and were considered as siderophore-producing isolates. The siderophore production was 23.74 psu by EC3B-22 and 71.8 psu by EC3B-23 (Table 2).

#### 3.3.5. Organic Acid Production

Table 2 lists the endophytic isolates, exhibiting their capacity to produce organic acids by a plate assay. A yellow zone development around the colonies was indicative as positive for organic acid production. Both the fungal and bacterial isolates were able to produce organic acid by showing the yellow zone development around the colonies. Production of organic acid resulted in a drop in broth medium pH, and fungal isolates EC1F-15 and EC3F-23 reduced the pH to 4.7 and 4.5, respectively, and bacterial isolates EC3B-22 and EC3B-23 reduced the pH to 5.1 and 5.0, respectively. 

#### 3.3.6. Extra Cellular Enzyme Assay

All the endophytic isolates showed diverse enzymatic activity. Fungal endophytes EC1F-15 and EC1F-23 exhibited amylase and lipase activities, while EC1F-15 was also positive for urease activity. Similarly, fungal isolate EC1F-23 was negative for urease but it exhibited tannase, gelatinase, protease, and phytase activities. Furthermore, endophytic bacterial isolate EC3B-22 was positive for gelatinase, protease, and phytase activities, while EC3B-23 was positive for amylase, lipase, and phytase activities (Table 3).

#### 3.3.7. Stress Tolerance of Zinc Solubilizers

All fungal and bacterial endophytic isolates were further assessed for their stability at different pHs, temperatures, and NaCl concentrations, and their optimum growth conditions were recorded. It was observed that isolate EC1F-23 grew well in the pH range between 5.0 and 10.0, while EC1F-15 preferred a pH of 8.0 for optimum growth. With regards to temperature, fungal isolates EC1F-15 and EC1F-23 showed better growth in the range of 25–35 °C. Both fungal isolates showed good growth in a diverse range of NaCl concentrations, from 0.5% to 3%, while EC1F-23 showed growth at 5 and 6%. None of the fungal endophytes showed growth at NaCl concentrations of 7–12%. Furthermore, the bacterial endophytes grew well in the pH range between 4.0 and 8.0, while little growth was observed even at pH 9.0. On the other hand, it was found that both bacterial isolates were able to grow well at temperatures between 15 °C and 35 °C, EC3B-23 grew also at temperatures of 40 °C and 50 °C. EC3B-22 showed restricted growth at 40 °C. EC3B-22 and EC3B-23 both grew well at NaCl concentrations ranging between 0.5% and 3% (Table 4).

#### 3.3.8. Characterization of Promising Isolates

**Figure 1:**
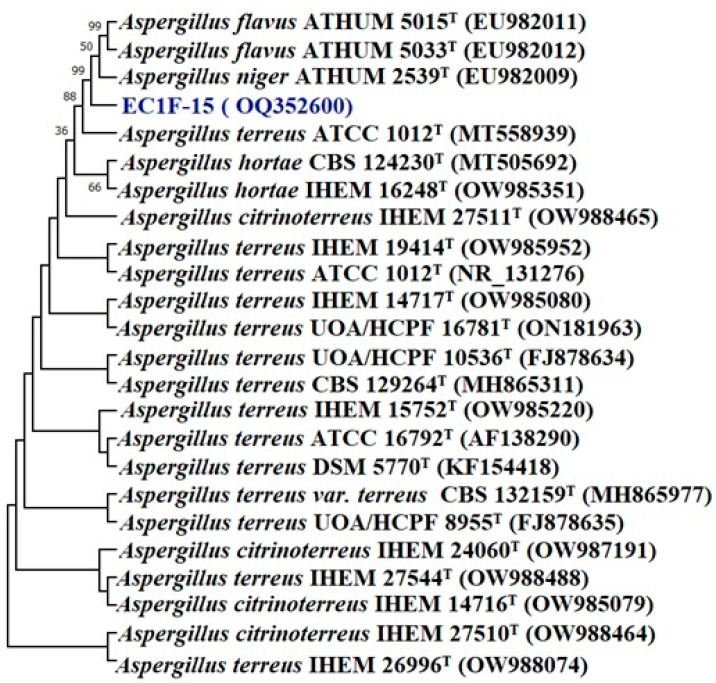
Phylogenetic tree of isolate *Aspergillus* sp. EC1F-15 obtained by maximum likelihood method based on the Kimura two-parameter model.

Based on the colony and spore chain morphology of fungal isolates, EC1F-15 and EC1F-23 belong to the genus *Aspergillus* and *Lecanicillium*. Based on the pairwise sequence alignment of ITS sequences, the isolates EC1F-15 and EC1F-23 share a sequence similarity of 99.18% and 99.82% with *Aspergillus terreus* 2011F5^T^ and *Lecanicillium* CCF 5201^T^, respectively. A phylogenetic analysis with type strains of the most closely related species of *Aspergillus* revealed that the isolate EC1F-15 is grouped in the clade formed by *Aspergillus niger* and *Aspergillus flavus* (Figure 1).

**Figure 2:**
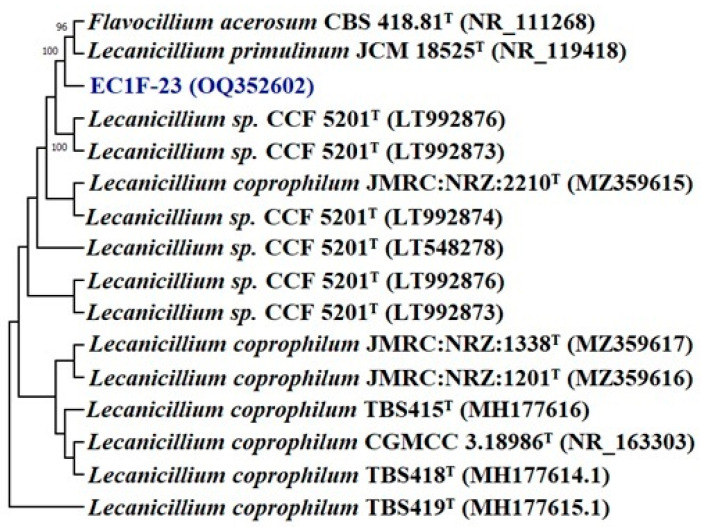
Phylogenetic tree of isolate *Lecanicillium* sp. EC1F-23 obtained by maximum likelihood method based on the Kimura two-parameter model.

This is supported by a high bootstrap value of 99%, which indicates it may be a new species of *Aspergillus*. Similarly, in the case of *Lecanicillium*, it forms a separate clade from *Flavocillium acerosum* and *Lecanicillium primulinum*, which is supported by a bootstrap value of 100%, indicating a new species of *Lecanicillium* (Figure 2).

**Figure 3:**
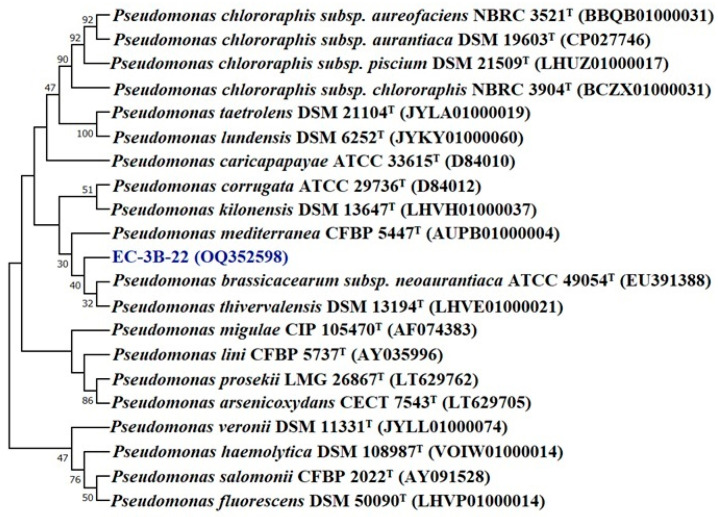
Phylogenetic tree of isolate *Pseudomonas* sp. EC3B-22 obtained by maximum likelihood method based on the Kimura two-parameter model.

**Figure 4:**
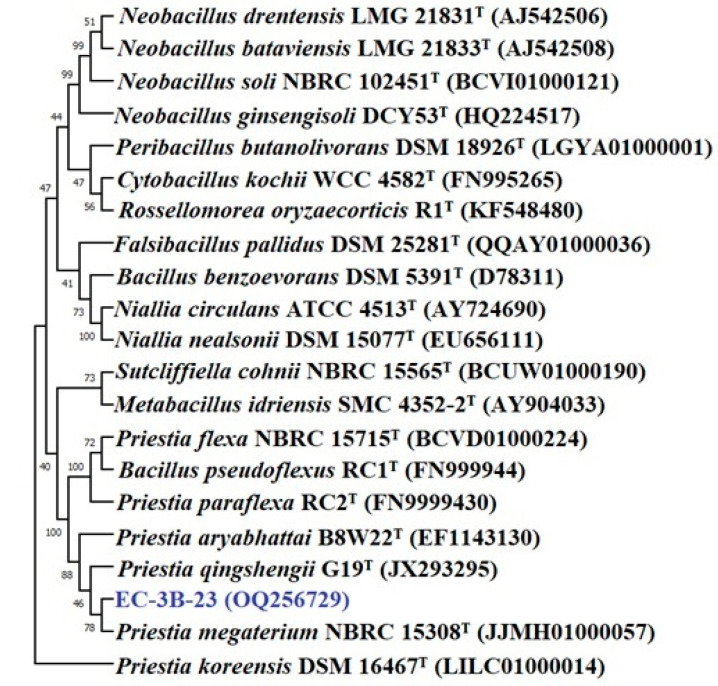
Phylogenetic tree of isolate *Priestia* sp. EC3B-23 obtained by maximum likelihood method based on the Kimura two-parameter model.

The bacterial isolates were identified based on a 16S rRNA gene sequence analysis. The pairwise sequence alignment (EZbiocloud database) of isolates EC3B-22 and EC3B-23 showed a sequence similarity of 99.85% and 99.51% with *Pseudomonas bijieensis* L22-9^T^ and *Priestia megaterium* NBRC 15308^T^, respectively. The phylogenetic analysis revealed that isolate EC3B-22 is in a different group to other species of *Pseudomonas* (Figure 3); hence, EC3B-22 isolates may belong to a new species. Isolate EC3B-23 is closely related to *Priestia megaterium* NBRC 15308^T^ in the phylogenetic tree (Figure 4).

The effects of various carbohydrates and nitrogen sources on the growth and development of endophytic fungi and bacteria were also examined. Endophytic fungi showed good growth on all the nitrogenous compounds, while the bacterial isolates did not show any growth on ammonium thiocyanate and ammonium vanadate. Both the fungal endophytes utilized all carbon sources, but EC1F23 did not show growth on mannitol, trehalose, and mannose (Table 4). Interestingly, bacterium EC3B-23 did not show any growth on galactose, arabinose, rhamnose, ribose, trehalose, mannose, and dextrin, which indicates that these sugars were not at all utilized by this bacterium (Table 5).

#### 3.3.9. Plant Experiment

On the basis of zinc salts, especially the zinc carbonate solubilization capacity, two fungal and two bacterial endophytic isolates were selected for plant growth and zinc mobilization experiments.

All four endophytes exhibited phytohormone production, phosphate solubilization, siderophore production, and various extracellular enzyme production abilities. The finger millet plants inoculated with fungal and bacterial endophytes were harvested after 90 days of inoculation in pot experiment conditions.

#### 3.3.10. Plant Growth Parameters

The shoot and root lengths were influenced by the inoculation of fungal and bacterial endophytes. In a plant growth experiment, the results of a pot trial showed that all isolates significantly improved the root and shoot parameters (shoot root length and dry weight) as compared to control plants. A significant surge in plant height and root length was observed in the finger millet cultivar VL-352 after inoculation with endophytic fungal isolate EC1F-23 (*Lecanicillium* sp.) and bacterial isolate EC3B-23 (*Priestia* sp.). Th maximum plant and root dry weight was recorded for EC1F-23-inoculated finger millet cultivar VL-348.

All three finger millet varieties showed higher plant height and root length com-pared to the control and ZC treatment. In variety PRM1, the maximum plant height was observed with bacterial isolate EC3B-23 (66.8 cm), followed by EC3B-22 (65.1cm). Similarly, inoculation of both the endophytic bacteria enhanced the root length (20.1 cm and 19.8 cm, respectively) compared to fungal endophyte ECF-15 (18.4 cm and 17.3 cm), the control (12.7 cm), and ZC (16.8 cm) treatments. In variety VL-348, the same trend was observed, where the maximum plant height (67.2 cm) was shown by EC3B-23, followed by EC3B-22 (64.8 cm), which was higher than the fungal-inoculated treatments (62.9 cm and 62.4 cm) and the uninoculated control. The highest root lengths (21.3 cm and 19.5 cm) were observed in EC3B-23 and EC3B-22 inoculated treatments, which was higher than the fungal and control treatments.

**Figure 5:**
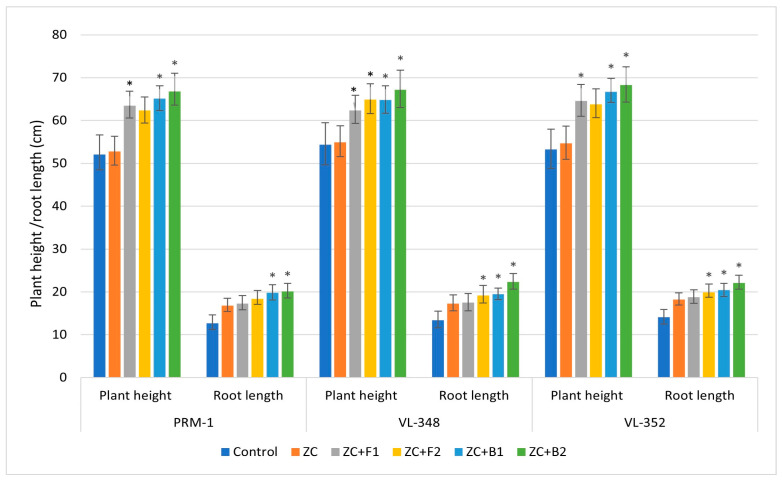
Endophyte inoculation influencing plant height and root length (error bars indicate the mean value of triplicate experiments ± standard deviation). (ZC—zinc carbonate; F1—EC1F-15; F2—EC1F-23; B1—EC3B-22; B2—EC3B-23.) * Indicates significate values at 5% probability.

Variety VL-352 interestingly also exhibited the same trend; bacterial isolates EC3B-23 and EC3B-22 displayed the highest plant height, i.e., 68.3 cm and 65.7 cm, respectively, followed by 63.8 cm and 63.6 cm by fungal isolates EC1F-23 and EC1F-15, respectively. VL-352 plants inoculated with bacterial and fungal isolates showed a taller plant height compared to the control (53.1 cm). The maximum root length of 22.1 cm was observed with EC3B-23, followed by EC3B-22 (20.4 cm), which was again higher than the fungal inoculated experiment, i.e., 19.9 cm and 18.8 cm (Figure 5). Significant increases in plant height and root length were observed after inoculating with endophytic fungi (EC1F-15 and EC1F-23) and bacteria (EC3B-22 and EC3B-23) as compared to the uninoculated control in all the three varieties.

**Figure 6:**
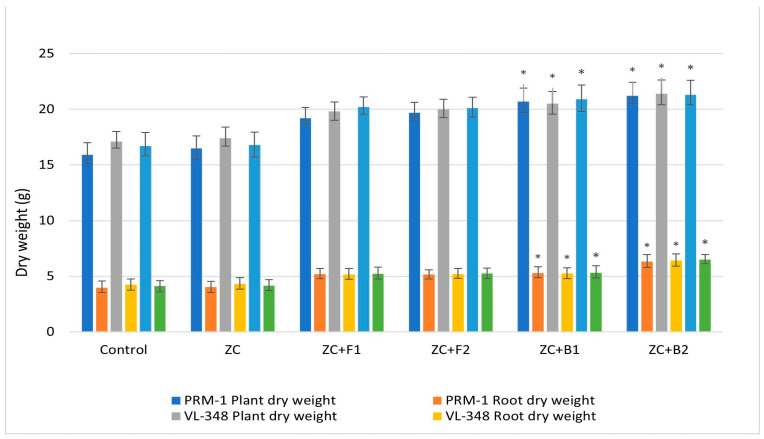
Endophyte inoculation influencing plant and root dry weights. Error bars indicate the mean of triplicate experiments ± standard deviation. (ZC—Zinc carbonate; F1—EC1F-15; F2—EC1F-23; B1—EC3B-22; B2—EC3B-23.) * Indicates significate values at 5% probability.

Regarding the plant and root dry weights, variety PRM-1 showed the highest plant dry weight at 21.2 g and 20.7 g after EC3B-23 and EC3B-22 treatment, respectively, which was higher than EC1F-23 and EC1F-15 treatment (19.7 g and 19.2 g, respectively). All four treatments showed higher plant dry weights compared to the control (15.9 g) and ZC (16.5 g). The same trend was observed in the root dry weight, where the highest root dry weight (6.31 g) was observed after EC3B-23 treatment. A similar pattern was also observed in varieties VL-348 and VL-352, where bacterial treatment (EC3B-23 and EC3B-22) resulted in the maximum plant and root dry weights, which were higher than both the fungal isolate and control treatments (Figure 6). The endophytic bacterial (EC3B-23 and EC3B-22) inoculated plants showed enhanced growth parameters which were significantly higher over the uninoculated control treatment.

#### 3.3.11. Zinc Content

**Figure 7:**
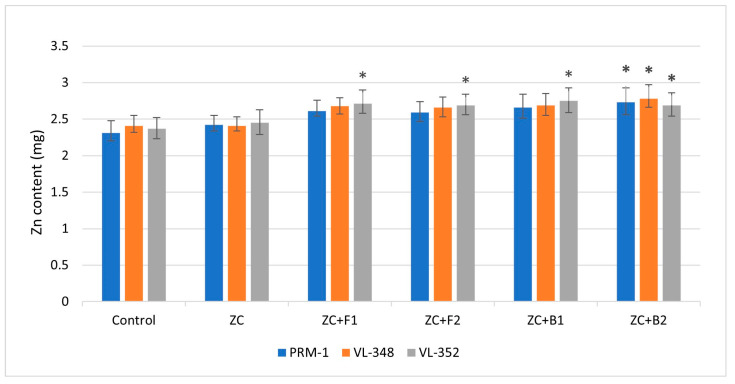
Endophyte inoculation influencing grain zinc contents. Error bars indicate the mean of triplicate experiments ± standard deviation. (ZC—Zinc carbonate; F1—EC1F-15; F2—EC1F-23; B1—EC3B-22; B2—EC3B-23.) * Indicates significate values at 5% probability.

The zinc content in grains was also influenced by endophytic inoculation; compared to the control treatment, the inoculated treatments showed higher zinc contents. In the PRM-1 variety, maximum zinc content (2.73 mg) was observed after treatment with EC3B-23, which was 18.8% higher than the control. Endophytic bacteria EC3B-22 inoculation resulted in a grain zinc content of 2.66 mg, which was 15.15% higher than the control. Similarly, inoculation of both the fungal isolates resulted in 12% more zinc content in grains compared to the control. The grain zinc content in treatment with ZC alone showed 4.76% more zinc content than the control. In the VL-348 variety, the highest grain zinc content was again observed after EC3B-23 treatment (18.80% higher than the control), followed by 2.68 mg after EC1F-15 treatment, which was 14.5% higher than the control. The other bacterial and fungal isolates resulted in 13–14% more grain zinc content compared to the control. In the VL-352 variety, the maximum zinc content was shown after treatment with bacteria EC3B-22 (2.75 mg), which was 18.02% higher than the control. The rest of the other microbial treatments showed 15–16% higher zinc contents in grains compared to the control (Figure 7). Significant increases in grain zinc contents were observed in the VL-352 variety after treatments with ZC + F1, ZC + F2, and ZC + B1. Treatment of ZC + B2 showed significant increase over control in all varieties.

The finger millet zinc content analysis showed that plants can uptake available forms of zinc through their roots and its transportation to the shoots can be facilitated by biopriming the plants with endophytes with plant-growth-promoting potential. These efficient endophytes colonize inside the plants and play an important role in zinc mobilization from root to grain via the shoots.

#### 3.3.12. NPK Concentrations

The NPK concentrations in grains were also significantly influenced by endophytic inoculation compared to the control treatment; the inoculated plants showed higher content. 

**Figure 8:**
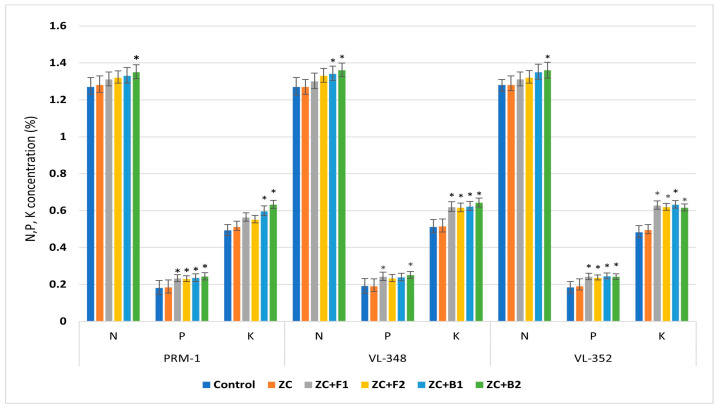
Endophyte inoculation influencing grain NPK concentrations. Error bars indicate the mean of triplicate experiments ± standard deviation (ZC—Zinc carbonate; F1—EC1F-15; F2—EC1F-23; B1—EC3B-22; B2—EC3B-23.) * Indicates significate values at 5% probability.

In the variety PRM-1, the N content was significantly higher over control after ZC + B2 treatment, the P content was significantly higher than the control in all the four endophytic treatments, while the K content was significantly higher after endophytic bacteria ZC + EC3B-22 and ZC + EC3B-23 treatments over uninoculated control treatment. For the variety VL-348, the N content was significantly higher than the control after endophytic bacterial treatment only, while the P content was significantly higher after treatments with ZC + F1 and ZC + F2, and the K content was significantly higher in all four endophytic treatments over the control. In variety VL-352, the N content was significantly higher after ZC + B2 treatment, while the P and K contents were significantly higher in all the endophytic treatments over control (Figure 8).

## 4. Discussion

Zinc is one of the main constituents of plants and is very important for their growth and metabolic activities. Deficiency of Zn is the most common micronutrient inadequacy in crop plants all over the world, resulting in considerable shortfalls in crop yields. Application of inorganic Zn fertilizers may not be economic in improving zinc insufficiency and enhancing crop yield [56]. Additionally, the application of Zn fertilizers is commonly practiced in several countries, including India, in spite of the extensive occurrence of Zn-deficient soils. These Zn-deficient soils hinder the normal growth of finger millet as well as principal foods such as wheat [57], rice [58], corn [59], and vegetables [18]. The foremost focus of this research was to isolate and characterize endophytic fungal and bacterial isolates from *Eleucine coracana* (finger millet) plants to prospectively dissolve Zn and be employed as Zn-biopriming agents to enhance the Zn content in finger millet, major nutrient uptake, and plant health.

Endophytic microbes possess the ability to dissolve zinc and are said to have a positive impact on plant growth and therefore they could be an alternative to chemical zinc fertilizers [60]. These microbes are of agronomic interest because they have been suggested as agricultural inoculants [61,62]. Endophytic isolates in this study were examined for their capacity to dissolve zinc in a medium supplemented with insoluble zinc salts [30]. The results revealed that two fungal and two bacterial endophytic isolates had varying solubilization potential with various insoluble zinc salts. The presence of a halo zone around the fungal and bacterial colonies on a solid medium was a sign of their zinc solubilization capability; the solubilization index (SI) varied from 3.0 to 4.8 mm for zinc carbonate, 2.9 to 5.6 for zinc oxide, and 2.1 to 3.1 mm for zinc carbonate.

The results showed that the chosen endophytic isolates exhibited diverse solubilization effects with different insoluble Zn salts. Several studies have established that fungal and bacterial isolates can dissolve zinc using different zinc sources with diverse results [17,26]. Dissimilarity among the microbial isolates is normal when several diverse microbes are examined for solubilization of Zn, and the solubilization efficacy outcomes showed that not a single isolate exhibited consistent effectiveness on all three salts of Zn. The highest dissolution of Zn was seen in zinc oxide, followed by zinc carbonate and zinc phosphate. These outcomes are in agreement with [26,63], which is opposed to [64], who showed the highest dissolution for zinc phosphate. The pH reduction was also accompanied by Zn solubilization, which could be associated with the endophytic production of organic acids such as gluconic acid, oxalic acid, citric acid, etc. [17]. In our study, a decline in pH was also observed when Zn salts were inoculated with endophytes, this result indicates that there is a relationship between the solubilization of Zn with reduced pH owing to the production of organic acids. The capability of endophytes to dissolve all three Zn salts is fascinating, since their presence in soils is known to occur in distinctive forms of chemicals with diverse solubility and accessibility to crops. Zinc plays a very significant role in plant growth enhancement and development; hence, perusing the plant growth parameters was another feature of the study. In our study, the endophytes were employed for their plant-growth-augmenting features and their effects on the mobilization of Zn from soil, Zn deposition in grains, and NPK nutrient uptake were monitored. 

Finger millet plants inoculated with endophytes showed relatively higher NPK concentrations in seeds compared to uninoculated plants. This suggested that finger millet plant inoculation with Zn solubilizing endophytes could have augmented the NPK concentration in plants compared to the uninoculated treatment [65]. This is obvious from the enhanced NPK grain content after inoculation with Zn solubilizing endophytes. This increase in N content could be accredited to the production of ammonia, nitrogen fixation by bacterial endophytes, and N mobilization by endophytic fungi. While the increases in P and K contents could be due to the production of organic acids and siderophores by the inoculated endophytes [66,67].

The selected zinc-dissolving endophytes also produced indole acetic acid phytohormones in amounts ranging from 35.4 µg/mL to 117.2 µg/mL. There are reports that propose that phytohormone-producing endophytes play a significant role in plant growth enhancement [68,69] and stress management [70,71]. 

On the basis of molecular analyses, two fungi, *Aspergillus terreus* and *Lecanicillium* sp., and two bacteria, *Pseudomonas bijieensis* and *Priestia megaterium*, were identified. Various species of *Pseudomonas* and *Bacillus* have been reported to produce IAA [72,73] and HCN [74], and also dissolve phosphates [75,76]. However, reports of endophytes from finger millet dissolving zinc were not found. 

Our studies are in accordance with the previously reported literature for zinc solubilizing bacteria, i.e., *Pseudomonas oleovorans* in [77] and *Priestia aryabhattai* in [78], and showed encouraging results. However, the interaction of all four endophytic isolates, as reported in this study, has not been conveyed with *Eleusine coracana* (finger millet) crops. 

The enhanced zinc content in wheat grains compared to the uninoculated plants has also been mentioned in previous reports [17]. Plant inoculation with endophytes resulted in improved plant growth and nutritional content; therefore, several isolates have been bioformulated as biofertilizers [79]. In addition to augmented plant growth, zinc-dissolving endophytic isolates used in this study also significantly enhanced the zinc and major nutrient contents in seeds compared to uninoculated plants; this has been extensively documented and discussed in several research works [22,80]. Previous studies have demonstrated that plant-growth-promoting endophytes enhance zinc translocation in maize [81], *Capsicum annuum* L. [82], chickpea (*Cicer arietinum* L.) [83], rice [23], and wheat [84]. This ability of endophytes to promote plant growth is owing to their capacity to induce successive plant–microbe interactions such as physiological processes, salt solubilization, and mineralization. Studies have shown that several physiological changes are triggered in plants when supplied with different chemical fertilizers or through biofertilization, which aids in the uptake of nutrients from the soil [80,85].

In addition to zinc solubilization, all endophytic isolates used in this plant–microbe interaction experiment were able to dissolve phosphate and produce IAA and ammonia. Further production of HCN by isolates could be another step towards using these microorganisms as biocontrol agents in addition to bioinoculants. Ref. [86] isolated an endophytic fungi *Fusarium* sp. from finger millet, which produced antifungal natural products. Again, in 2016, [87] reported that root endophytic *Enterobacter* sp. inhibits *Fusarium graminearum*, a finger millet fungal pathogen, by creating a physiochemical barrier. Similarly, [88] reported the endophytic structural and functional diversity in finger millet and reported that endophytes *Bacillus cereus* and *Paenibacillus* sp. are efficient at inhibiting the growth of blast disease causing pathogens in finger millet. Therefore, finger millet endophytes also exhibit biocontrol potential in addition to mineral mobilization and plant growth promotion. 

Zinc solubilization by plant-growth-promoting endophytes is a relatively novel approach and most of the isolates have not been tested on *Eleusine coracana* (finger millet). This study indicates the potential of fungal endophytes *Aspergillus terreus* and *Lecanicillium* sp. and bacterial endophytes *Pseudomonas bijieensis* and *Priestia megaterium* to be used as potential bioinoculants to overcome zinc deficiency in countries such as India, where zinc fertilizers are not cost-effective and are under used. The employment of these endophytes in finger millet for plant growth promotion and Zn biofortification has not been reported previously. However, field trials are required to determine the commercial usability of the studied endophytes so that they can be established as plant growth promoters and Zn mobilizers for their commercial utilization in finger millet cultivation.

## 5. Conclusions

In this study, zinc-dissolving fungal endophytes *Aspergillus terreus* and *Lecanicillium* sp. and bacterial endophytes *Pseudomonas bijieensis* and *Priestia megaterium* were isolated from finger millet varieties. Besides dissolving zinc, these endophytes were also able to dissolve phosphate and exhibited various plant-growth-promoting properties, such as phytohormone, siderophore, HCN, and ammonia production. All four endophytes were able to enhance the uptake and transportation of zinc from the soil to the grains. This was evident from the production of organic acids and acidification of zinc media, creating a halo zone around the colonies. Inoculation of finger millet plants with these endophytes increased the shoot and root lengths and the plant and root dry weights, along with increasing the zinc content and NPK content in grains compared to uninoculated controls. These endophytes have shown efficient capabilities to dissolve insoluble zinc compounds such as carbonate, phosphate, and oxide, which makes them a valuable source for enhancing zinc uptake and fortification in seeds, NPK uptake, and the growth of finger millet crops.

## Figures and Tables

**Table 1:** Zinc salts and phosphate solubilization by endophytes.

Isolates	Zinc Solubilization			Phosphate Solubilization
	Zinc Carbonate	Zinc Oxide	Zinc Phosphate	
	SI (mm)	SI (mm)	SI (mm)	SI (mm)
**Fungal isolates**				
EC1F-15	3.0 ± 0.63	2.9 ± 0.52	2.6 ± 0.49	4.3 ± 0.74
EC1F-23	3.3 ± 0.65	3.6 ± 0.61	3.1 ± 0.53	3.6 ± 0.68
**Bacterial isolates**				
EC3B-22	4.3 ± 0.58	3.4 ± 0.71	2.1 ± 0.35	2.4 ± 0.63
EC3B-23	4.8 ± 0.61	5.6 ± 0.82	2.3 ± 0.49	2.5 ± 0.56

**Table 2:** Siderophore, IAA, HCN, ammonia, and organic acid production by endophytes. The pH was measured at the time of organic acid production.

Endophytic Isolates	Siderophore Production		IAA Production (µg/mL)	HCN Production		Ammonia Production	Organic Acid Production	pH
	Qualitative	Quantitative Percent Siderophore Unit (psu)		Qualitative	Quantitative (OD)			
**Fungal isolates**								
EC1F-15	+	63.7 ± 0.54	35.4 ± 0.46	++	0.031 ± 0.002	−	+	4.76 ± 0.13
EC1F-23	+	60.6 ± 0.48	82.4 ± 0.74	−	0.009 ± 0.001	−	+	4.54 ± 0.18
**Bacterial isolates**								
EC3B-22	+	23.74 ± 0.60	117.2 ± 0.67	+++	0.043 ± 0.002	−	+	5.16 ± 0.12
EC3B-23	+	71.8 ± 0.63	105.5 ± 0.59	++	0.029 ± 0.001	+	+	5.04 ± 0.16

**Table 3:** Extracellular enzymes produced by endophytes.

Isolates	Enzymes						
	Amylase	Lipase	Urease	Tannase	Gelatinase	Protease	Phytase
**Fungal isolate**							
EC1F-15	+	+	+	−	−	−	−
EC1F-23	+	+	−	+	+	+	+
**Bacterial isolate**							
EC3B-22	−	−	−	−	+	+	+
EC3B-23	+	+	−	−	−	−	+

**Table 4:** Diverse abiotic stress stability is shown by endophytes.

Endophytic Isolates	Fungal Isolates		Bacterial Isolates	
	EC1F-15	EC1F-23	EC3B-22	EC3B-23
**Growth at different pHs**				
pH 4.0	+	+++	++	++
pH 5.0	++	+++	++	++
pH 6.0	++	+++	+++	+++
pH 7.0	++	+++	+++	+++
pH 8.0	++	+++	+++	++
pH 9.0	+	+++	+	+
pH 10.0	+	+++	−	−
**Growth at different temperatures**				
5 °C	−	−	−	−
15 °C	++	++	+++	+++
25 °C	+++	+++	+++	+++
30 °C	+++	+++	+++	+++
35 °C	++	+++	++	+++
40 °C	+	+	+	++
50 °C	−	+	−	++
**Growth at different NaCl concentrations**				
0.5%	+++	+++	+++	+++
1%	+++	+++	+++	+++
2%	+++	+++	+++	+++
3%	++	+++	+++	+++
4%	++	++	−	−
5%	−	++	−	−
6%	−	+	−	−
7%	−	−	−	−
8%	−	−	−	−
9%	−	−	−	−
10%	−	−	−	−
11%	−	−	−	−
12%	−	−	−	−

**Table 5:** Growth of endophytes on different nitrogen and carbon sources.

Isolates	Fungal Isolates		Bacterial Isolates	
	EC1F-15	EC1F-23	EC3B-22	EC3B-23
**Nitrogen source**				
Peptone	+++	+++	+++	+++
Yeast extract	+++	+++	+++	++
Beef extract	+++	+++	+++	+++
Ammonium bromide	+++	+++	+++	+++
Ammonium thiocyanate	+++	+++	−	−
Ammonium persulphate	+++	+++	+++	+++
Ammonium vanadate	+++	+++	−	−
Ammonium bicarbonate	+++	+++	+++	+++
Ammonium acetate	++	+++	++	+++
Ammonium molybdate	+++	+++	+++	+++
Ammonium dichromate	+++	++	+++	+++
Ammonium ferrous sulphate	+++	+++	+++	+++
**Carbon source**				
Glucose	+++	++	+++	+++
Fructose	++	++	+++	+++
Lactose	++	+	+	+++
Inositol	+	+	+++	+++
Maltose	++	++	+	+++
Malt extract	+++	+++	+++	+++
Sorbitol	+	++	+++	+++
Mannitol	++	−	+++	+++
Galactose	+++	+	+++	−
Arabinose	+	+++	+++	−
Rhamnose	++	++	+	−
Ribose	++	++	−	−
Trehalose	++	−	−	−
Sucrose	+++	+++	+++	+++
Mannose	++	−	+++	−
Dextrin	+++	+++	+	−

## Data Availability

All original contributions presented in this study are included in the article, further inquiries can be directed to the corresponding authors.
